# Medicine shortages in Fiji: A qualitative exploration of stakeholders’ views

**DOI:** 10.1371/journal.pone.0178429

**Published:** 2017-06-05

**Authors:** Josephine Walker, Betty B. Chaar, Numa Vera, Alvish S. Pillai, Jessy S. Lim, Lisa Bero, Rebekah J. Moles

**Affiliations:** 1 Faculty of Pharmacy, University of Sydney, Sydney, Australia; 2 College of Medicine, Nursing and Health Sciences, Fiji National University, Suva, Fiji; 3 Charles Perkins Centre, University of Sydney, Sydney, Australia; Jagiellonian University, POLAND

## Abstract

**Objectives:**

Medicine access is a human right; yet, concerningly, there are international instances of shortages. Quantitative data has allowed WHO to propose global solutions; however, individualised understanding of specific regions is still required to work towards national solutions. Fiji has an established issue with medication supply and the aim of this study was to use qualitative methods to gain a fuller understanding of this context.

**Methods:**

Semi-structured interviews were used to gain the perspective of key stakeholders involved in the Fijian medicine supply chain in regards to causes, impacts and possible solutions of medicine shortages. Thematic analysis was used to analyse the interview data.

**Results:**

In total, 48 stakeholders participated and the information was synthesised into three main themes, causes, impacts and solutions and the sub-themes including; political, system and patient causes, adverse health effects on patients, professional dissatisfaction, monetary loss and loss of faith in the health system, workarounds, operation improvements, government intervention and education and training.

**Conclusions:**

The situation in Fiji is not dissimilar to other instances of shortages around the world and hence international solutions like that proposed by WHO are feasible; however, they must be modified to be uniquely Fijian to work in this context.

## Introduction

Medicine is crucial to healthcare and hence considered a human right [1]. This has been characterised by the World Health Organisation’s (WHO) Essential Medicines List (EML) which includes items that manage basic health concerns and disease burden [2].

Medicine shortages however, are a globalised phenomenon [3–7], with a long history of occurrence [6, 8]. They result in inconvenience and/or profit losses and pose a health risk to patients due to inadequate, interrupted, or lacking treatment [6, 9]. Evidence suggests that shortages have been increasing [4, 9], causing global concern [6].

Quantitative research reveals many causes related to supply and demand [6, 8]. ‘Supply’ includes the impact of natural disasters [4], manufacturing problems [8] and financial issues [4, 8]. ‘Demand’ is attributed to increased use of medicines as in outbreaks/ epidemics, “just in time inventories”, and the effects of brand competition/marketing [4, 6, 8]. Global solutions suggested by WHO include shifting focus to patient-centred care, increases in communication and training, reshaping the global market to increase production and involvement of not-for-profit organisations (NGOs) to create a worldwide accessible buffer stock [6]. Discussion has also involved viable pricing of medicines, universal quality control and creation of national/international reporting systems [6].

However, medicine shortages are diverse and manifest differently [3, 4]. Quantification allows for better understanding on a global scale, however, understanding the intricacies of individual instances of shortages and their consequences are lacking [8]. Qualitative data provides in-depth insight to supplement numbers with a human aspect, focusing on the social impacts of the given issue [10].

**Fig 1:**
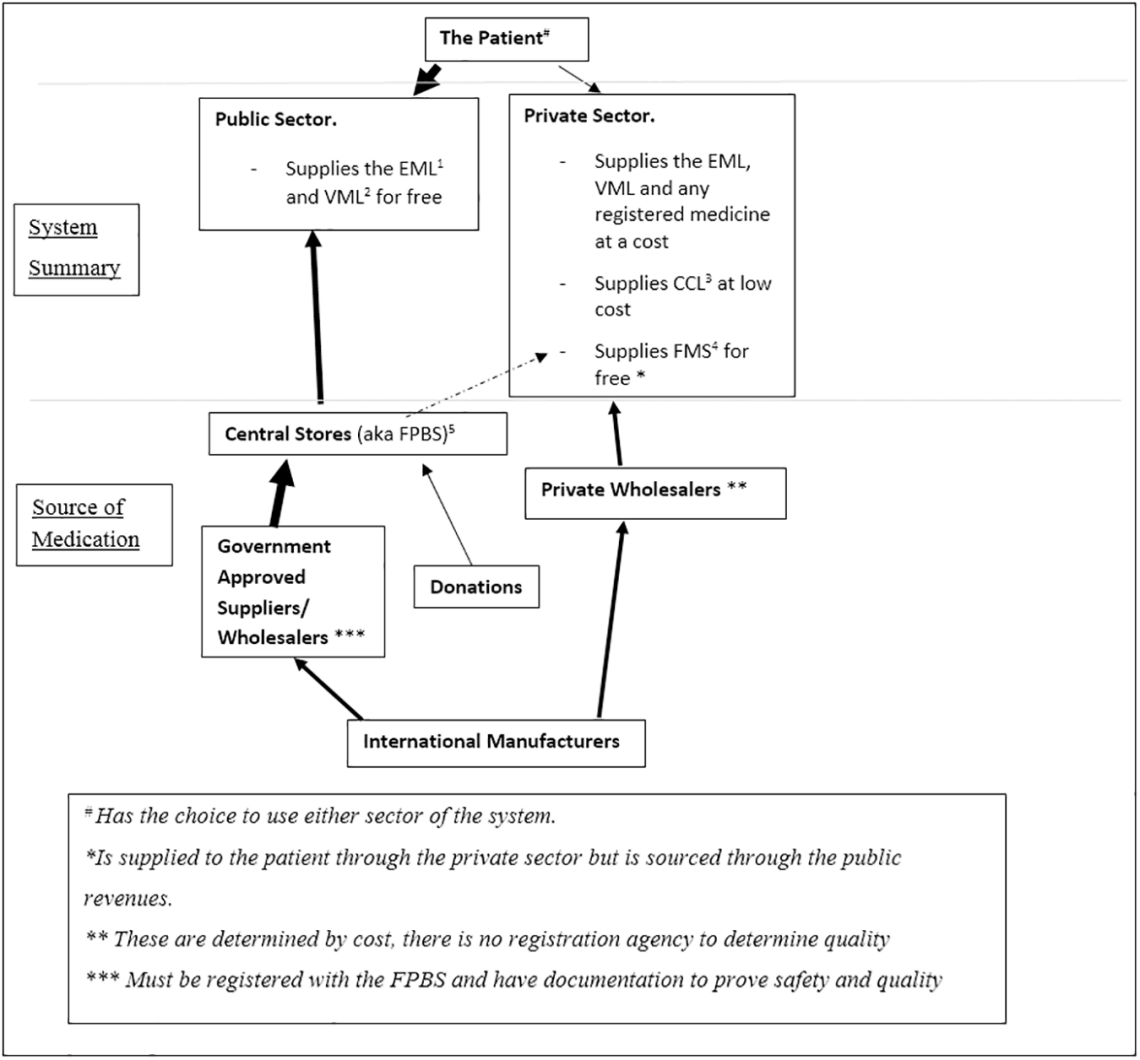
A summation of how patient’s access medicines within the Fijian healthcare setting [14]. (The arrows represent movement towards an area, whether this be people or medicine. The thicker arrows indicate heavier use of this pathway.). Additional explanation of the different aspects can be found in Table 1.

Fiji is an island nation in the South Pacific, home to approximately 850,000 people [11]. In a developing nation like Fiji [12], shortages are often associated with increased risk of morbidity and mortality [5]. The Pacific Island Countries are a health-conscious area of the world, with a substantial focus on public health; however non-communicable diseases (NCD) now pose a threat to human, social and economic aspects of this region [13]. Fiji has both private and public healthcare sectors (Fig 1 and Table 1) [12, 14], and an established problem with medicine access [12, 15]. In 2004 the WHO/HAI survey tool was used to ascertain accessibility and affordability of essential medicines [12]. Public sector data was not reported [12] but it was found, across private pharmacies, that 23.3–26.7% of medicines were not available to patients [12]. This was considered satisfactory, but a range of possible improvements were suggested, including installing a quality assurance system and patient education [12]. Medicines were considered affordable to an employed person, so cessation of free outpatient supply was also suggested [12]. Finally, it was concluded that the health budget could not support the importation of the entire EML–hence refinement was proposed [12].

**Table 1:** The common medicine access schemes in Fiji.

Abbreviations	
^1^ Essential Medicines List (EML) [16]	A list comprising of medicines considered essential for the management of priority health conditions. This is created based on the WHO list but has had additions made which are relevant to healthcare in Fiji. These medicine are considered safe, effective and often low cost. (Full list available at http://www.health.gov.fj/wp-content/uploads/2015/06/EML-4th-Edition.pdf)
^2^ Vital Medicines List (VML) [16]	This is a list of medicines which are used in life or death situations or those which are essential and have no alternative treatment. There is a distinction made between essential and vital medicines in Fiji, though technically the vitals are a subset of the EML. (Full list available at http://www.health.gov.fj/wp-content/uploads/2015/06/EML-4th-Edition.pdf)
^3^ Free Medicines Scheme (FMS) [17]	A list of essential medications supplied as lowest price generics supplied as a set price in the public sector which is considered affordable but also supports economic growth and development (Full list available at http://www.commcomm.gov.fj/wp-content/uploads/2012/05/Revised-Determination-Quarter-1-2014.pdf)
^4^ Commerce Commission List (CCL) [18]	This is a list of 72 (soon to be 142) essential and non-essential medication which is being made available free in the public sector to those who meet the low income criteria. They are supplied as the lowest priced generic, sourced and supplied by the Ministry of Health. (Full list available at http://www.health.gov.fj/wp-content/uploads/2015/03/Free-Medicines-Program-Leaflet.pdf)
^5^ Fiji Pharmaceutical and Biomedical Services (FPBS)	Is a government body that coordinates the acquisition and distribution of medicine for the public sector.

Generally, Fiji lacks reputable information into medicine availability and utility. The aim of this study was to build on current knowledge of medicine shortages in Fiji, focusing on the causes, impacts and possible solutions through a qualitative lens to gain a fuller understanding.

## Methods

Qualitative research was conducted in Suva, Fiji from July through October 2016. Semi-structured interviews were conducted with stakeholders involved in the medication supply chain. A standardised interview-protocol was used, based on available literature and studies objectives, adapted from a similar Australian-based study [19] (S1 File).

### Ethics

Ethical approval was gained for this study from the relevant institutions: the Fiji National Health Research Ethics and Review Committee, Ministry of Health and Medical Services Fiji, and the Human Research Ethics Committee of the University of Sydney [2016/719].

### Data collection

Individuals were approached for interviews based on experience in the field, using purposeful sampling from both the public and private sector. This included pharmacists, doctors, nurses, logistics managers and bureaucrats. Individuals who agreed to participate then signed formal consent forms. Interviews were audio-recorded using “Smart Recorder” on a smart phone. Data collection continued until all aspects of the supply chain, excluding consumers’ perceptions, were covered and data saturation occurred.

### Data processing and evaluation

Recordings were transcribed verbatim, subsequently analysed using thematic analysis [20] and core themes extracted using NVivo 11.

## Results

**Fig 2:**
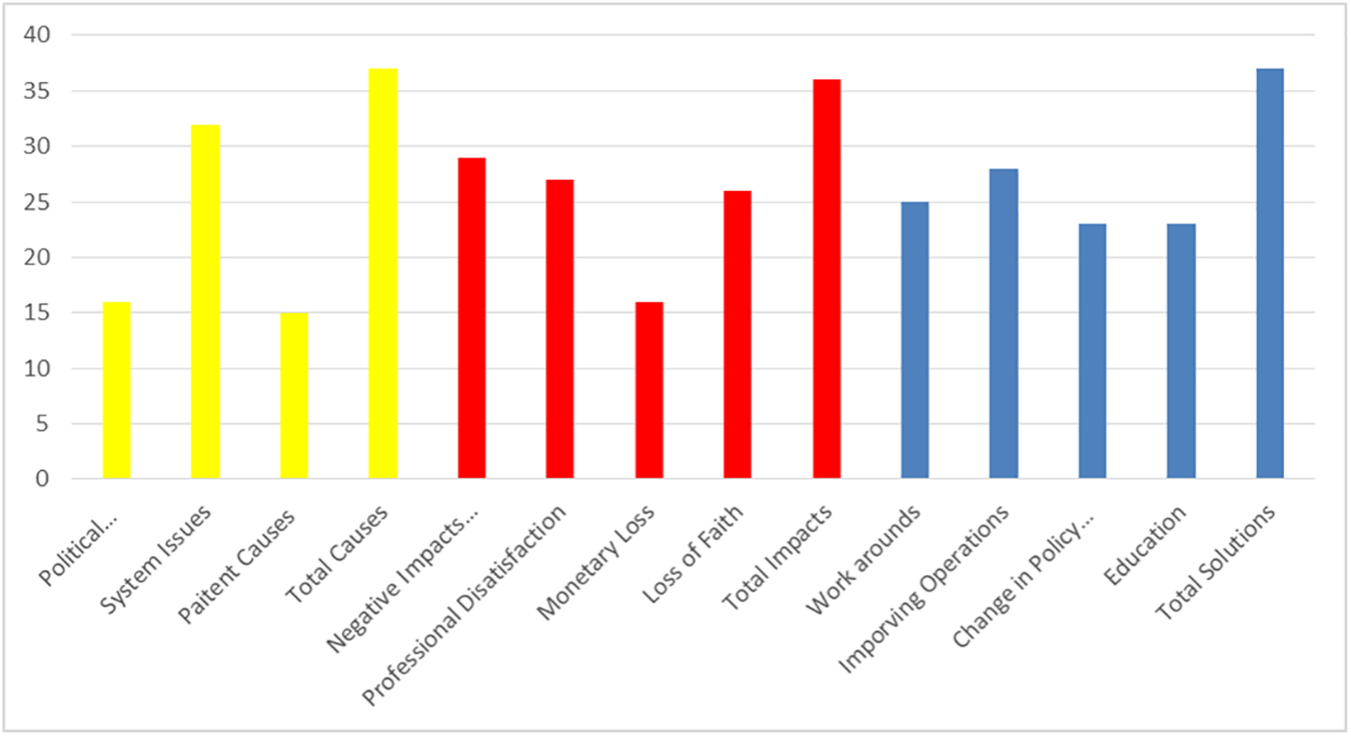
A summary of topics discussed by 48 participants in 37 interviews. The yellow bars indicate the causes, red the impacts and blue the solutions.

**Fig 3:**
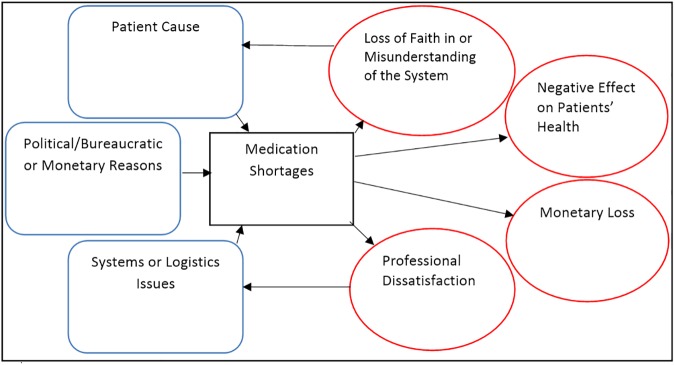
Summation of the causes and impacts of shortages expressed as an influence diagram. The arrows represent the influence one aspects holds over another.

A total of 48 people were interviewed in 37 interviews including 17 pharmacists (5 operating in retail pharmacies, 12 in the public sector), 9 doctors (2 in private practice, 7 in the public sector), 13 nurses and 9 bureaucrats/policy makers. Average interview time was 13 minutes and ranged from 4–48 minutes. From the information compiled, three major themes were extracted: causes, impacts and solutions. Each theme was then broken down into sub-categories (Fig 2) and interactions between the different aspects explained (Fig 3).

### Causes

#### Political/Bureaucratic or monetary reasons

Stakeholders reported issues acquiring stock due to complete reliance on international sources. The small buying-power meant stock came from suppliers rather than manufacturers. This often increased prices without guaranteeing supply.

“For us small countries, coming in with small money and small quantities, we are actually probably in a queue at the manufacturing level.” [Interview11]

A capped budget, kept consistent in recent years despite increased medicine costs and country requirements, also exacerbated the situation. Decreased finances increased the time needed to organise tenders and meant sea/land-based transport. This caused slower transport and longer durations of stock-outs. Lack of financial support also limited human resources and motivation.

“So the total request for this year was twelve million, except you’ve got to work in nine million” [Interview18]

“The bureaucracy just takes forever… to get some approval from some person who’s probably… not really doing their job” [Interview22]

Cost also affected the private sector. The CCL had set prices and due to lack of profit, private buyers opted to keep limited or no stock of these medicines. Also, very few suppliers could meet the set price, which again increased the risk of stock-outs.

“Let’s say Commerce Commission already has a set price. And let’s say if I were to import a drug and if I can’t meet their price that means I wouldn’t be importing that drug because I can’t sell that below cost.” [Interview26]

Finally, the public sector only had access to medicines on the EML and the VML. This primarily meant that stock was effective and affordable. However, it created another barrier to stock acquisition of items no longer considered first line globally, like tetracycline eye drops, resulting in fewer suppliers.

“We still use those drugs that have become obsolete in Australia and New Zealand. So finding those becomes an issue, causing a huge shortage.” [Interview2]

#### Systems issues or logistics

The most prevalent system issue identified was poor quantification. There was no consistent system to monitor the usage of stock and wastage, which translated to compromised forecasting. The communication between the health centres and the government buying agency, FPBS was variable and inconsistent.

“There’s a big disconnect in the organisational side of the medications organisation; there does not seem to be a really good idea of predicting the changing need. I’ve asked for records of what the previous amount of the drugs used is and those records aren’t available” [Interview27]

Procurement was another issue. As identified by respondents, government priorities currently lie with providing services rather than the procurement of medicines, giving the impression that more is then being done in healthcare improvement. There was a lack of trained professionals in charge of ordering, and it was often reported to be “too late” to cater for the long transport times. Faster travel options were only considered within budget if stock was completely out.

“We’ve got a cardiac centre where we do stenting… But they don’t have a very good supply of stents. They don’t have a regular supply of the medication that is required to keep them working. But as far as certain people are concerned they have ticked that box.” [Interview19]

“We really lack staff in Fiji, especially specialised in logistics” [Interview29]

The private sector kept limited EML stock of generic essential medicines as patients accessed these mainly from the public sector. Poor communication between the sectors however meant that when it was out of stock in the public sector, a product was rarely ordered in the private sector to ensure continuity of care.

“If it’s on the EDL (Essential Drug List) but people are not buying it, I don’t think we go that extra step to try to procure it.” [Interview25]

#### Patient causes

It was reported that the mindset of the public has been changing with a greater reliance on medicines to fix all ailments and hence a greater number of visits to hospitals for treatment. Some stakeholders reported a lack of resources to sustain this. It was also reported that many individuals also obtained multiple prescriptions in short amounts of time and abused the EML and FMS to hoard medicines, thus adding to the short-fallings in public stock. Interviewees suggested that people also misunderstood where they could access free medicines. Patients often preferred traveling to hospitals to source medicines, and were unaware they could access free medicine from local health-clinics, causing hospital shortages and inappropriate distribution. Further, some misunderstood the importance of adherence to chronic therapy, had resistance to changed brands or, felt they could not afford to buy medicines from retail pharmacy.

“They would only get what’s available at the hospital. If it’s unavailable, they’ll just go without it.” [Interview26]

“If they get discharged without their medications, they don’t go outside and buy their medications. Some, can’t afford, some just are not bothered” [Interview8]

### Impacts

#### Negative effect on patients’ health

Medicine shortages were causing ill-health. Interviewees reported that shortages resulted in longer hospital stays, shorter times to patient readmission and even mortality.

“Sometimes we have to stop the operation because of the shortage” [Interview17]

“Thrombolytics were out of stock and then, all of a sudden, we had a lot of cardiac deaths … If you don’t thrombolyse, they come back with failure.” [Interview18]

“We’d like to make them live longer but then if drugs are out of stock we will not be able to do that.” [Interview24]

“Ventolin has been out of stock since June and we get asthmatics on the ventilator; we can’t treat them. Some people have died because we haven’t been able to treat them.” [Interview27]

#### Professional dissatisfaction

Medicine shortages resulted in appreciable levels of frustration, particularly for professionals involved. Patients were angry with pharmacists, prescribers and nurses, who reported violence and threats. Also, shortages meant that professionals had to dedicate extra time and effort to solving the issues; taking time away from patient care and other roles, such as inventory management. Many interviewees felt unable to meet the public’s expectations of a healthcare provider. This generated a negative workplace environment, extending even to a sense of hopelessness. This was reported in both public and private sectors.

“For me it comes down to what I can deliver for the patients and if you don’t have drugs and equipment then everything else becomes obsolete.” [Interview18]

“If we are unable to supply the needs of patients, we are obviously not doing our jobs.” [Interview6]

“We are humans, we feel for our patient and you want to give the best care, but we cannot provide the best care because of no medication…” [Interview31]

#### Monetary loss

Medicine shortages were causing financial loss in Fiji. Stakeholders reported the negative opinion the government held of medication supply because of the constant cost of medicine with limit returns in terms of decreased healthcare cost and consumer satisfaction.

“Talking to many major politicians, they look at the pharmacy and they say, it’s like a black hole, ever-wanting something.” [Interview19]

It was reported that any financial loss resulting from shortages was shifted from the government to the hospitals, private settings or directly to the patient. This impacted on patients’ livelihoods and health, if the cost prevented adherence.

“In the neonatal some of our babies need surfactant. One vial costs $1000!!!” [Interview35]

#### Loss of faith in or misunderstanding of the system

Interviewees reported that consistent shortages caused reduced trust in the public healthcare system. Similarly, there was discussion about the closed nature of the system, whereby consumers were not supplied with information about shortages and/or where they could access their medicine. Generally speaking, the health literacy of the Fijian people was perceived as low and to avoid confrontation they were often not provided with information on where they could access alternative supplies in case stock was unavailable there also.

“Certainly when you haven’t got option A, then you go B … do we tell patients? Of course not. Should we tell patients? Of course, yes. Why not? Because then people lose faith in the system.” [Interview21]

“We don’t really want to raise the expectations and then not able to meet them,” [Interview9]

Professionals also reported an acceptance of current shortages, conveying little hope of improvement. When looking at the system as a whole, this translated into stagnation in working towards solutions and, for some, the adoption of lax work-ethic.

“It’s like continuous and contagious” [Interview10]

“We have never had any time that, say, zero out of stock. It’s not possible.” [Interview12]

“It’s beyond our control” [Interview14]

“… F.P.B.S doesn’t have any stock, so we’ll take it at that. I mean, that’s all we can do.” [Interview31]

### Solutions

#### Current work-arounds

There were a range of solutions in place including: limiting stock to inpatient use, reducing the amounts given to outpatients and directing patients to source stock elsewhere. In procurement, the current work-around was to ensure the most important medicines were in stock and bear the consequences of not being able to supply others. Some nurses even reported keeping their own supplies of certain medicines to use for their patients.

“We will keep on reprioritising what we need to buy and we’ll keep buying vitals and the important ones, those we think aren’t important, we won’t buy” [Interview11]

“Sometimes we hide our supply. When we know we are running low, we hide it because the other ward will come and ask” [Interview32]

#### Improvement in operations

The operations system in Fiji was clunky and disconnected between different sectors. Interviewees suggested a complete overhaul. However, others expressed that components of a functional system were all existent in Fiji—the main concern was refining the system.

“What we need to do is probably work smarter, not work harder” [Interview9]

The most important factor to address was quantification, including the identification of appropriate wastage and buffer stocks. Participants stated that all aspects of the system needed uniting and the processes needed constant updating. There was an inclination to incorporate computers into the process, with the provision that training was provided and the limitations of the software were made clear before installation. These changes however were articulated to “not be possible without addressing the mindset of the people working within the system”.

“I think we if we have to make use of computers, then purchase can be linked everywhere,” [Interview14]

**“**Sometimes when people are used to doing things for years and years it’s very hard for them to change their culture and they develop particular culture and they’re used to it” [interview 28]

“One of the biggest problems has been that the system has become modernised but the mindset hasn’t caught up.” [Interview19]

#### Change in policy and/or government intervention

There was call for a change in government funding; particularly in re-evaluation of the EML and essential services. Evaluations should also be more frequent to capture current best practise. To achieve this there were assertions that more pharmacists, medicinal experts and people trained in basic logistics and stock management should be employed in evaluation and procurement to ensure the correct and viable stock was ordered.

“Without a proper definition of what is an essential service we are stuck.” [Interview19]

There was controversy over the FMS. Some individuals supported it with the provision of increased advertisement. Others felt policy changes were needed to force people to use it or otherwise should be abandoned all together to bring more medicine supply back into the public sector.

“To limit the free medication scheme to government health facilities only as plenty of the drug which have been circulated to private pharmacies gets expired and wasted.” [Interview15]

There was also discussion on shifting from a free to a paid organisation-funded system to help ease the burden on the government. This would include the introduction of an insurance scheme or reimbursement to the needy, which would need to be fully clarified before any shift could be made.

“I don’t think people realise how important it is to take their medications because it’s free. They need to take ownership of their lives so they need to fork out money because it will force them to pay attention.” [Interview33]

One individual also reported regionalisation as a way of overcoming Fiji’s lack of buying power

“we need to be part of a regional procurement program.” [Interview19]

#### Education and training

The most practical application of education believed to be increased training for those involved in inventory management, procurement and ensuring medicine quality.

“I think it may mean being able to sacrificing a scholarship overseas for engineering or something and look at Fiji Pharmaceutical Services.” [Interview21]

Additional it was reported that education for the general public was required. Measures like the CCL, FMS, and established insurance agencies, which would ease demand on the public system, were currently underutilised or perceived as too expensive; education has the potential to correct perceptions. Also, education on positive medication use, behaviours and lifestyle may give the system the reprieve it needs in order to rebuild and improve.

“I think there needs to be more education and awareness.” [Interview9]

## Discussion

This qualitative study explored key stakeholders’ views of perceived causes, impacts and proposed solutions of medicine shortages in Fiji, particularly in the public sector. Logistic failures were the most common reason for shortages, and impacts on both healthcare professionals and patients were highlighted by all participants. Solutions proposed by key stakeholders were practical, however barriers to implementation included resource deficits, both human and monetary. Furthermore, some stakeholders suggested shifting the “mindset” of bureaucrats, health professionals and even consumers may be required.

Many of the causes and solutions applicable in Fiji parallel those of the WHO publication addressing global medicine shortages [6]. Similarities in causation include reduced buffer stocks, hoarding practises, increased want for NCD treatment and manufacturers withholding tenders due to small order size [6]. One major issue highlighted was unreliable data from facilities outside major cities [6]. This was mentioned consistently by stakeholders in conjunction with poor quantification.

WHO states that improvement to medicine access includes employing supply chain management techniques with effective inventory management and evaluation of procurement [6, 21]. Most interviews indicated inadequate quantification and communication of stock levels at various facilities including FPBS, hospital pharmacy and rural settings. Stakeholders suggested two areas to address; human resources and accurate reporting/forecasting. There were concerns for the number of skilled healthcare providers in Fiji, particularly in rural clinics which are exclusively staffed by nurses. One commonly discussed solution was introducing logistics specialists into the system to establish the importance of this role and set a standard for medicine acquisition and reduce the work burdens of other healthcare professionals. Though, this was rebuffed by limited training opportunities. There is no formal logistics training available through Fijian universities. But, a small scale study by Wu Y-CJ et al (20), showed there were improvements in logistic based knowledge and skills in participants of online based logistic courses [22]. Provided access to computers and internet could be provided, these qualifications conducted via correspondence, could serve to help fill this learning gap until other Fijian based training opportunities can be developed. Another viable option could be providing incentives for training abroad. As revealed in a study by Mudalair LA, Fijian students aspire to study abroad as they can gain a greater amount of knowledge, particularly in utilisation of software and computers in industry, to bring home [23]. There are also scholarships available through the Fijian government to encourage this behaviour [23] and hence there is already a system that could be utilised to train a logistics specialist to help improve the siltation. However, in this setting pharmacists do undergo basic logistics training so task shifting is also a possible short-term solution that has been used in other countries [24]. Trained assistants could be brought in to aid in dispensing [24], freeing pharmacists to focus on stock control. Similarly, the pharmacists could be more involved in medicine procurement, product selection, distribution and quantification at FPBS because, whilst annual usage data exists there is a lack of workforce to assist in comprehending trends and forecasting. Another issue identified was wastage, particularly in rural centres. Adding flexibility into the system and adding provisions including a transport system to redistribute surplus medicines might also help increase efficiency. However success would rely on effective communication between and within health centres.

Stakeholders reported that information on stock status is often unclear and/or too late to employ conservative behaviours. Also, central authorities have concerns for incorrect reporting in peripheral healthcare. The information inconsistency is concerning. A contributing factor is the changing healthcare setting, where the system is becoming decentralised [25]. In fact, worsening rates of shortages have been reported since initiation of decentralisation in 2001 [25]. Centralised medication acquisition has shown beneficial results for medicine stock management in other settings [25, 26]. Also, WHO suggest a consistent definition of medication stock-outs and a nation-wide reporting system is preferable [6] A countrywide, computer-based system in Fiji has many potentials for improvements including; communication to distant healthcare [27, 28], data collection and synthesis [27], organisation within the supply chain [27] and possible improvements therefore to patient health [27, 28]. But, Fiji lacks resources, notably technology including computers and software in the healthcare system [28]. Previous attempts to “modernise” have created new problems including the inability to use the systems and inconsistent use, resulting in inaccurate stock levels and a hesitance to embrace change. Also, many developing nations have financial limitations [27]. However, building on current systems have the potential to reduce costs [28] and combinations of IT and manual records have been shown to be easier to install, cost effective and have a greater acceptance [27]. But, it is crucial that installation, like any change, is done in coordination with a well-structured plan and time frame [25, 27] with continuous training of those involved [27].

Stakeholders reported concerns for transport times post ordering, caused by using cost-friendly, Asia-based suppliers and order-sized based restrictions on suppliers. There is evidence to suggest that smaller nations have the capacity to gain medicines at a reduced cost, which could be utilised by Fiji [29]. And there was discussion about coordination with overseas organisations/nations to build the acquisition powers of Fiji. There are successful examples of this, including the Pan-American Health Organization which operates on behalf of different South American countries and uses the increased numbers to gain stock more efficiently than the countries could do independently [30]. A revolving fund means countries reimburse the fund for the items they use after procurement [30]. A similar program was run by the formation of a group of the six Persian Gulf states [30]. These systems have saved costs and increased and maintained vaccine supply for these two groups. However, the success of the programs is dependent on the cooperation of the nations involved, a consensus on the type and quantity of medicines and transparency in the ordering process [30]. As expressed above, Fiji would need to improve quantification before this could be possible and, the Fijian formulary would have to updated to match that of other nations involved. Also, there are concerns for cooperation within the region. There have been many attempts to regionalise the South Pacific [31]. The most recent, the Pacific Plan, has lost appeal because of undeveloped cooperation and negative economic aspects [31]. Also, exclusive trade agreements between Australia and New Zealand neglect other nations preventing free trade discussions in the region [31]. This solution addresses the biggest issues in medication acquisition and should not be disregarded. But, foundation work needs to be done before these solutions become reality. As expressed by stakeholders, agreements with off-shore hospitals could assist in the acquisition. Also, many NGOs may allow access to medication through buying bulks of medication and reselling at cost, like the GAVI Alliance [32]. This could serve as a temporary means to ease the burden of medicine shortages in Fiji until permanent trade agreements are established.

One WHO recommendation is that medicines should be a viable price for the supplier and consumer [6]. In the private sector, Fiji has focused on the latter through the CCL [18] and FMS [17]. Both include EML items, utilising lowest priced generics so medicines are affordable for the general public [18] and free to those with a lower income [17]. These projects have worth, especially in low-middle income countries [33] where generic use has an estimated cost saving of 60% [34]. However, evidence suggests these programs can be damaging to retail pharmacy without correct management [33]. As interviews suggested, Fiji has already taken steps to ensure the system is viable by using more than three suppliers to minimise potential for stock-outs [33]. However, the system uses a set profit gain of 5% [18] opposed to a price cap. This limits possible profit and acts as a deterrent for pharmacists to dispense these items [33]. These issues were iterated by stakeholders, especially when their own generic stock was considered reasonably priced. There were also concerns for the viability and possible benefits of these programs. There were reports of low consumer uptake of the CCL because of lack of awareness and mistrust of generics; but also pharmacist’s resistance to dispense these items due to concerns of quality. Historically there has been evidence of counterfeit medicine importation [12] but this has been overcome through testing conducted through the FPBS and professional awareness [34, 35]. There is a wealth of information on bioequivalence and availability of generic medicines [36–43]. This has the potential to be used in patient education to shift usage habits [34, 35] and overcome barriers. Also, prescribers should be educated and encouraged to inform their patients on the use of generics as this may increase the use of generics [44]. Retail pharmacists held negative views of the FMS because of a complicated system, the physical space requirements, and the lack of finical benefit. Studies reveal monetary incentives for professionals can increase uptake of generic medicine programs [34]. Again, this could be a way for government to support these programs and help ease stock shortages in the public sector.

An interesting finding was the human impacts resulting from shortages. There is professional dissatisfaction because of continued stock-outs. This is a significant impact that needs to be addressed considering the shortage of healthcare providers and the tendency to seek international employment. Healthcare provider demotivation can also negatively impact the way individuals are able to access health care [45] and therefore should not be overlooked. Fiji is a dynamic healthcare setting [25] and professional empowerment, in the form of training, has the potential to promote expertise, skills and improve attitudes [46]. Also, if done in appropriate areas such as logistics training and non-pharmacological disease-state management, post-registration training could contribute to reducing the burden of shortages. However, forcing individuals to partake in extra training with an expectation that they fund this can have negative effects [47] hence incentives to continue education would be part of this solution. System improvement is only made possible by addressing all aspects, including the people.

Stakeholders held concerns for patients’ perceptions of health, including late presentation to hospital, dependence on medicines opposed to lifestyle changes, reduced personal responsibility for their health, hoarding, potential overuse of medicines and poor adherence to chronic therapy. Lacks in health literacy has been shown to worsen health outcomes, especially in developing nations, and adds to the burden of medicine shortages [48]. In the wake of Fiji’s growing prevalence of NCD’s [13] there is a growing need to involve people in their treatment to help tackle to the above issues. Shared decision making could assist in aligning treatment with an individual’s values and capabilities [49]. Also, it increases patient knowledge [49], which is important in appropriate medication use and developing help-seeking behaviours, and trust between prescribers and patients [49]. In addition, increasing the availability of reliable information on medicines, like the “wise list” a Swedish guide on commonly prescribed medication designed for consumer use, increases patient knowledge but also adherence and appropriate use of medicines [50]. One suggestion made by stakeholders was to charge fees for medicines and healthcare in order to place more value on treatment and prevent overuse of the system. This may be viable, but must be done with caution if at all. There are correlations between costs of medicines and low adherence, hence price could act as a barrier to healthcare [51]. Considering a significant proportion of Fiji’s population live below the poverty line [52] any cost might be considered too high for many individuals. However, more research into patients’ perceptions of healthcare and the causes and impacts of shortages in Fiji is required.

Also important is reported impacts on patient health such as increased morbidity and mortality. This has been reported extensively in relation to medicines shortages [53–55], however the realities of these impacts emphasises the importance of this topic and the need to further research from a consumer perspective.

This is the first qualitative study of its nature in the region, which we are aware of, and was strengthened by the broad range of stakeholders involved. However, as with any qualitative study the results hold some biases and preconceived ideas of the interviewer. Furthermore medicine supply is considered a government issue and means that this topic is often considered controversial which may have prevented open discussion. However, as a third party, non-Fijian researcher conducted the interviews and participants were reassured of their anonymity, it was felt that many participants were able to express their true opinions. Patients were not approached in this study hence the lack of firsthand, patient perspective may act to limit the findings, and presents a topic for future investigation.

## Conclusion

It can be concluded that medicine shortages are a significant, ongoing issue within Fiji. Although complex, the situation is similar to global occurrences. Hence, there is much that can be learnt from international solutions such as investment in logistics training and human resources, the introduction to a nationwide reporting system, regional support, education and training. However, to be fully functional these solutions must be adapted to find an answer that is uniquely Fijian.

## Supporting information

S1 File Interview protocol. This is the interview protocol that was used in the interviews conducted in this study.
(PDF)
