# Supplementary material for: Medicine shortages in Fiji: A qualitative exploration of stakeholders’ views
Source: PLoS One. 2017 Jun 5;12(6):e0178429. doi: 10.1371/journal.pone.0178429 (PMC5459560; doi:10.1371/journal.pone.0178429)
Supplement: S1 File — This is the interview protocol that was used in the interviews conducted in this study. (PDF) [file pone.0178429.s001.pdf]

# Interview Protocol

**Medication Shortages: Impact and Management Strategies**

Step 1

Introduction and greeting

Step 2

Reiteration of participation options (withdrawal etc), organise consent form signing and ask for permission to start audio-taping.

If participant is in a remote area and the interview is via telephone:

Particular care will be taken in acquiring the particpant's express consent [written already received prior to the interview] on the record in accordance with the relevant legislation [Telecommunications Act 1997 (CTH) and Listening Devices Act 1984 (NSW)] which prohibit recording of phone interviews without the express consent of all parties

Step 3

Start interview- allow participant to speak without prompts. Use prompts only if the flow lulls somewhat.

INTERVIEW QUESTIONS

1. What are your general thoughts about medication shortages?

    Prompts:

    a. Do you experience medication shortages in your practice?

    b. How often do shortages occur in your practice?

    c. What do you know about causes of medication shortages?

    d. What are the implications and effect on practice of medication shortages?

2. What is the role of the essential and Vital Medicines Lists in your country?

    Prompts:

    a. How does a medicine becoming included on the list?

    b. When do you refer to the list?

    c. How is the list used? (reimbursement, procurement etc)

    d. Do consumers know about the list and its use?

3. What do you believe could be done to improve the current situation?

Thank the participant and ask if they wish for feedback in the form of a summary of findings.
